# Supplementary material for: Rural populations exposed to Ebola Virus Disease respond positively to localised case handling: Evidence from Sierra Leone
Source: PLoS Negl Trop Dis. 2020 Jan 21;14(1):e0007666. doi: 10.1371/journal.pntd.0007666 (PMC6994168; doi:10.1371/journal.pntd.0007666)
Supplement: Attachment — Submitted filename: Reviewers comments PNTD-D-19-01217.docx [file pntd.0007666.s002.docx]

**Reviewer’s comments**

**Manuscript number**: PNTD-D-19-01217

**Manuscript title:** Rural populations exposed to Ebola Virus Disease respond positively to localised case

handling: evidence from Sierra Leone

**General comments**

Thanks kindly for the opportunity to review this paper. The research employs qualitative methods to assess community perceptions regarding the introduction of decentralized Ebola Community Care Centers during Sierra Leone’s Ebola outbreak of 2014-2015. The research was conducted in February 2015 – several months after the introduction of the CCCs, at a time when infection levels were starting to wane but when recall/perceptions of the community regarding the approach were likely to still be recent and strong enough to generate robust data. The research demonstrated high levels of acceptability of the CCC model and an overall positive response to the intervention.

The CCC approach was controversial at the time of introduction – with questions of safety and effectiveness frequently raised. The model was a major departure from the established approach of high-tech Ebola Treatment Units (ETUs) which were far from communities and where patients often presented late in their illness.

The paper is well researched and well written – with a substantial sample of respondents drawn from villages where CCCs were housed, as well as villages where they were not. The research accurately depicts community concerns regarding ETUs – and their potential to drive the disease further underground and prolong transmission. The ‘complementary’ nature of these facilities within the wider Ebola response is well articulated. The findings are also consistent with previous research.

**Specific comments**

**Introduction**

1. Line 92 – may want to note that Ebola holding centers had no IPC standards and likely fueled transmission. Patients were free to interact with one another.
2. Line 111 – the sentence reads that the analysis seems to unpack ‘why communities say CCC in a positive light’. Suggest being more circumspect here and rather state that the research aimed to explore community perceptions – positive and negative, regarding the CCC approach
3. Line 112 – seems you are profiling findings from the research in the introduction ‘CCCs matched cultural expectations’ etc. Similar to statements around land tenure. Should save this for results/discussion
4. The end of the introduction should clearly state the aims of objectives of the research.

**Methods**

5. Setting – may want to include something about the context/ setting of where the CCCs were established ie. Rural, remote, poor transport access in general, far from the capital and ETUs, high-transmission areas
6. May want to mention that PHC facilities were not felt to be sufficiently ‘protective’ / IPC standards to house Ebola patients. May also want to cite the fact that many villages were afraid to use PHC centers due to fear of contracting Ebola. Some CCCs were located on the PHC grounds – and were intended to allow more ‘routine’ cases (non-Ebola suspects) to more safely use the PHC facilities
7. The selection process of ‘matched control villages’ is unclear to me. Was this 1:1 ratio with the CCC villages? How were they chosen? Were these villages intended to be served by the CCCs (it seems so)? Also, I am not sure the term ‘control’ villages is appropriate – may opt for ‘CCC resident’ vs ‘ CCC referral’ villages.
8. Line 132 – how were respondents recruited? Were they from existing social networks (churches, women’s groups)?
9. Were the FGDs recorded and translated? Was qualitative software used for analysis?
10. Ethics – Unusual not to get IRB approval. It’s definitely ‘primary research.’ Did the research have government permission?
11. Line 191 says all subjects were adult, but in line 133 you suggest there were FGDs amongst youth?

**Results**

12. Line 202 – The description of the CCC model would better sit at the beginning of the Methods section
13. Line 224 – you mention distance to ETUs as an issue. You may want to note how far and how long it would take to get there (perhaps in the setting section of the methods)
14. The research didn’t mention anything on the village-based CCC approach fostering any changes related to improved awareness of Ebola and community ownership of the response. Did this come out in the findings?
15. Line 294 – may want to note that families were not allowed to oversee burial independently as the ritual washing of corpses was a major factor driving Ebola transmission
16. Lines 319/321 – correct bye-law to ‘by-law’
17. Line 347 – on the issue of acquiring land – no fees were paid for the use of land. should note that the CCCs were intended to be temporary structures. The village chief negotiated placement of the centers in each village with the community based on a set of specifications – flat, access to water/road, etc. The land was returned to the community at the end of the response once the CCCs were decommissioned
18. There is nothing in the results section on perceived differences in responses between CCC villages and controls. Were there any differing perceptions?

**Discussion**

19. Line 382 – you note ‘good road accessibility’ – however much of Sierra Leone, particularly where CCCs were located, were far from the tar road network. Port Loko might be an exception – but Bobali and Tonkolili were quite remote
20. Line 393 – might want to cite previously published data with figures on the shorter time to presentation at CCCs relative to ETUs (ref 2)
21. Line 427 – note comment 15 above
22. Line 435 – please highlight the point in comment 17 above
23. Line 468 – you should use reference 2 to cite the data on transmission effects
24. Finally – the paper doesn’t really cite research from elsewhere on the central role of affected communities in taking ownership of responses for other infectious diseases – HIV, TB, vaccine preventable disease. Including this in the discussion would strengthen the paper
