# Supplementary material for: Rural populations exposed to Ebola Virus Disease respond positively to localised case handling: Evidence from Sierra Leone
Source: PLoS Negl Trop Dis. 2020 Jan 21;14(1):e0007666. doi: 10.1371/journal.pntd.0007666 (PMC6994168; doi:10.1371/journal.pntd.0007666)
Supplement: Attachment — Submitted filename: PNTD-D-19-01217 Rebuttal.docx [file pntd.0007666.s003.docx]

| The Netherlands

PLOS NTD

Dear editors,

Thank you for giving us the opportunity to revise our paper titled "Rural populations exposed to Ebola Virus Disease respond positively to localised case handling: evidence from Sierra Leone" (#PNTD-D-19-01217). Many thanks also to the reviewers for their profound comments. We are very pleased by the positive recommendations.

We have addressed all the comments, leading to an improved manuscript as submitted. In the tables below we indicated how we responded to the detailed comments of each of the reviewers.

For the general comments we would like to thank reviewer #1 for the expressed appreciation of our manuscript. We very much agree with the way reviewer #1 stated the relevance of our paper. General comments of reviewers #2 and #3 are well received. These mainly highlight or summarize the detailed comments and are addressed as indicated below.

We look forward to your decision on the revised version.

With kind regards,

Harro Maat, also on behalf of Esther Yei Mokuwa

**Reviewer 1**

| **Comments**  **Introduction** | **Responses** |
| --- | --- |
| Line 92 – may want to note that Ebola holding centers had no IPC standards and likely fueled transmission. Patients were free to interact with one another | Agreed, but the passage simply aims to summarise the debate in the UK parliamentary Committee on Overseas Aid, and we see no easy way to insert the point, without (wrongly) implying it was part of the debate in the House of Commons |
| Line 111 – the sentence reads that the analysis seems to unpack ‘why communities say CCC in a positive light’. Suggest being more circumspect here and rather state that the research aimed to explore community perceptions – positive and negative, regarding the CCC approach | Agreed. We have used the suggested reformulation. |
| Line 112 – seems you are profiling findings from the research in the introduction ‘CCCs matched cultural expectations’ etc. Similar to statements around land tenure. Should save this for results/discussion | This passage has been deleted. It is covered later in the text. |
| The end of the introduction should clearly state the aims of objectives of the research | Added |
| **Methods** |  |
| Setting – may want to include something about the context/ setting of where the CCCs were established ie. Rural, remote, poor transport access in general, far from the capital and ETUs, high-transmission areas | Yes, points added |
| May want to mention that PHC facilities were not felt to be sufficiently ‘protective’ / IPC standards to house Ebola patients. May also want to cite the fact that many villages were afraid to use PHC centers due to fear of contracting Ebola. Some CCCs were located on the PHC grounds – and were intended to allow more ‘routine’ cases (non-Ebola suspects) to more safely use the PHC facilities | Yes, points added |
| The selection process of ‘matched control villages’ is unclear to me. Was this 1:1 ratio with the CCC villages? How were they chosen? Were these villages intended to be served by the CCCs (it seems so)? Also, I am not sure the term ‘control’ villages is appropriate – may opt for ‘CCC resident’ vs ‘ CCC referral’ villages. | Good suggestion. We have changed the text accordingly, and clarified selection criteria |
| Line 132 – how were respondents recruited? Were they from existing social networks (churches, women’s groups)? | As now further explained in text |
| Were the FGDs recorded and translated?  Was qualitative software used for analysis? | Each FGD had two facilitators, one tasked with transcribing and translating comments as they were made. No recording equipment was used because we considered this would inhibit people in making frank comments.  No. The data were coded by the first author and a second person independently, according to criteria now mentioned in the introduction to the paper. Results were compared and reconciled. |
| Ethics – Unusual not to get IRB approval. It’s definitely ‘primary research.’ Did the research have government permission? | Yes, the work was requested by DFID and MoHS. There was careful liaison with local authorities and community representatives. Note in text is amplified. Context is explained in a further detailed note to editors, attached |
| Line 191 says all subjects were adult, but in line 133 you suggest there were FGDs amongst youth? | Youth is a category of adulthood in rural Sierra Leone (typically includes young adults up to age 35, but at times older, when of low social status). We did not include any children. Now explained in the text. |
| **Results** |  |
| Line 202 – The description of the CCC model would better sit at the beginning of the Methods section | We agree. |
| Line 224 – you mention distance to ETUs as an issue. You may want to note how far and how long it would take to get there (perhaps in the setting section of the methods | Good idea. Done. |
| The research didn’t mention anything on the village-based CCC approach fostering any changes related to improved awareness of Ebola and community ownership of the response. Did this come out in the findings? | Yes, but only by inference from a fuller contextual analysis, for which there is no room in the current paper. |
| Line 294 – may want to note that families were not allowed to oversee burial independently as the ritual washing of corpses was a major factor driving Ebola transmission | True, but the point being made is that all SDBs were constituted by volunteers being given training; the point is why those volunteers could not more often have come from Ebola-affected communities. In one case we know of (Lower Banta chiefdom) the DERC agreed to allow the training of community volunteers after a team based in district HQ took several days to attend to a burial. We have amended the phrasing to make clear this was not a request for families to continue washing corpses in the traditional manner. This was widely understood by our informants. |
| Lines 319/321 – correct bye-law to ‘by-law’ | The official announcement approved on August 8^th^ 2014 used the spelling “Byelaws” We would prefer the Sierra Leone spelling (though with a hyphen) unless it is against your house style. |
| Line 347 – on the issue of acquiring land – no fees were paid for the use of land. should note that the CCCs were intended to be temporary structures. The village chief negotiated placement of the centers in each village with the community based on a set of specifications – flat, access to water/road, etc. The land was returned to the community at the end of the response once the CCCs were decommissioned | All true, but we are not sure it needs to be said here. The comments from the FGDs make it clear that the community understood it was their responsibility to offer the land free, for a limited period. The issue they mainly brought up was that no chief had the right to order them to give land. That would be disrespect. It had to be requested, and then given freely. |
| There is nothing in the results section on perceived differences in responses between CCC villages and controls. Were there any differing perceptions? | An interesting comment. The answer is few, and none of any statistical significance. The problem is how to report a negative result. We have added a brief passage to the discussion. |
| **Discussion** |  |
| Line 382 – you note ‘good road accessibility’  – however much of Sierra Leone, particularly where CCCs were located, were far from the tar road network. Port Loko might be an exception – but Bombali and Tonkolili were quite remote | not our view, but the view of MSF in its evidence to the UK parliament  Agree. So we have repeated the citation and clarified the point |
| Line 393 – might want to cite previously published data with figures on the shorter time to presentation at CCCs relative to ETUs (ref 2) | Yes, a good suggestion. We have done so. |
| Line 427 – note comment 15 above | noted |
| Line 435 – please highlight the point in comment 17 above | noted |
| Line 468 – you should use reference 2 to cite the data on transmission effects | done |
| Finally – the paper doesn’t really cite research from elsewhere on the central role of affected communities in taking ownership of responses for other infectious diseases – HIV, TB, vaccine preventable disease. Including this in the discussion would strengthen the paper | References are added. |

**Reviewer 2**

| **Comments**  **Introduction** | **Responses** |
| --- | --- |
| Aim of the study should be more clearly articulated – at the moment the authors have stated that these data were collected as ‘part of an assessment of the impact of CCC’ – could they provide more specific aims/objectives of this study and how it differs from the overall assessment of which this is a part? | This is now provided |
| **Methods** |  |
| Did all villagers participate  Unclear how participants were identified  This section would benefit from some additional detail | All villagers were invited, at a public meeting with the village head. Participation was voluntary.  They self-identified according to the age and gender categories mentioned at the introductory meeting. Everything was done with the authority and approval of Paramount Chief and village chiefs, since large spontaneous gatherings were prevented by chiefdom Ebola Bye-Laws  More detail has been supplied |
| Topic guides – were these semi-structured/unstructured | The guide was a list, but facilitators were asked to allow comments to flow as the group wished. They ticked off topics as they arose spontaneously, and added any important ones missing later in the interview, to assure broad comparability across all the FGs. The aim was to avoid leading questions as much as possible. |
| Who conducted the fieldwork? | The first author and her team. The team all had extensive experience working with her carrying our village household surveys and running focus groups in previous field research in Sierra Leone since 2010. |
| What level of training did they receive? | Extensive. She previously held a research grant from 3IE to develop and evaluate FGD methods in rural Sierra Leone. The protocol for the CCC study came from this previous project, and all the field assistants were trained on it. |
| If the FGDs were conducted simultaneously, and each had two facilitators, does this mean the field team was comprised of 8 people? | Yes |
| What is meant by a ‘statement’ in the context of specifying the number identified? | As recognized by two coders working independently on FGD transcripts. |
| What is meant by ‘descriptive’ vs ‘evaluative’ statement types? | Now explained in the text. |
| What analytical approach was utilised?  How were themes developed and determined? | The two coders (first author and one other, who did not take part in the research, but with research knowledge of social impacts of Ebola) agreed a list of 12 themes of likely relevance after reviewing literature, and debate on the online Ebola Anthropology Research Platform, covering local responses to EVD. They then coded independently, and reviewed and resolved disagreements. The transcripts are available on line and are open to further independent coding. |
| Line 162-163 – unclear what differences (between villagers and health agencies) are explored and how the views of agencies are represented given that the data collection approach described here only focuses on interviews with community members. More detail on methods required. | We agree that the phrasing is regrettable. We are analysing only local responses, and not the views of health agencies. The sentence has been amended |
| Table 1 – not clear what this depicts. | It shows the numbers of speakers who made Type 2 (evaluative) statements on the four themes discussed in the paper. |
| Lines 169-181 – did the Douglas schematic serve as an analytical framework for the data? Make clearer. Why this approach rather than another? | It was chosen because Douglas offers clear a priori reasoning for approaching questions of risk and blame from the perspective of clashes of value between actors organized into hierarchies and egalitarian enclaves (a situation regularly encountered in Sierra Leone). But this part of the analysis belongs to a larger study. An earlier version of the paper included the discussion and justification the reviewer requests, but it made the paper unwieldy. This paragraph remains only to provide a pointer to this further work-in-progress. The paragraph has been amended to make it clear that the present paper does not attempt that analysis, but limits itself only to a descriptive account of the data set. |
| What steps were taken to manage vulnerability or distress? | Facilitators were trained to be aware of signs of distress, and to halt proceedings if this became an issue. All interviewers were mature and experienced persons sharing local cultural values, and capable of identifying local indicators. The second facilitator was tasked with observing the body language of participants and responding accordingly. Risks of vulnerability were reduced by not seeking to include former patients or survivors. All participation was voluntary |
| Could the collective approach to agreeing participation have risked that some participants felt compelled to participate?  What steps were taken to avoid this? | This is unlikely since the voluntary nature of the activity was clearly explained in the community meeting introducing the FGD process and ask if people wished to participate.  The steps of passing through correct channels for approaching communities and seeking their cooperation, including offering the gift through which strangers seek collective approval to ask questions (offering *famaloi*). Approval of the community at this point removes the obligation of those who do not want to speak to take further part, since the community as a whole has agreed to provide information. Those who wish to guide the visiting stranger correctly will then play their part. There is some possibility that this filters information, but it is not a coercive process. |
| **Results** |  |
| Line 207 “all CCC had a water supply etc…” – all CCC in the country or all the CCCs that were the focus of the research? If the former, please add references. CCCs visited by this reviewer were not fully equipped, although that might have been at an earlier stage in the outbreak. | Agree this should be clarified. Only the CCCs we studied. This is now changed in the text. |
| Very unclear how this distinction between descriptive and evaluative statements was determined. What was the analytical process here?  What is the significance of drawing this distinction? | Between a factual statement and an opinion. E.g. “our CCC had a water supply” and “The well at our CCC dried up because they dug it in the wrong place”.  We did not rely on the FGDs to generate factual information, only opinions about whether the CCCs were positively valued. The purpose of the research was to understand whether CCCs were accepted by communities. The text has been amended t make this a bit clearer. |
| Lines 222-227: what is the significance of the quantitative breakdown? E.g. male vs female – how does this relate to the gender split among participants? | The gender split is apparent in the fact that the two adult focus groups were divided into groups for men, and groups for women (thus approximately 50:50). |
| Did men express this sentiment more because it was more important to them (and if so, why?), or does this just reflect the demographics within the sample? | Yes, that is an implication from the numbers once the sample design is taken into account (having more or less equally populated groups for men and women). |
| Unless there is a very good reason for presenting qualitative data quantitatively, it is generally not good practice.  In this article it is not clear why this approach has been taken, or indeed how to interpret the quantitative data presented | We respect the referees purist preferences, but do not share them, preferring to work with mixed methods. Given a stratified sample design the numbers can be interpreted as a rough measure of the relative importance of different themes to the four different sample strata (high status elders versus low status youth, and adults stratified by gender).  It follows from the sample design. We believe it is important for the reader to grasp that the opinions to which we assign interpretive significance are not just outliers but are supported by some significant weight of opinion. |
| What is the significance of the number of times a particular issue was raised? What is the base here? How does it relate to the overall sample? | Again, the answer is the sample design, and a desire to avoid over-interpretation of outliers. The card tracking system allows the analyst to discover patterns of responses – e.g. whether certain opinions were favoured in some but not all of the four groups, whether certain people dominated the conversation, or whether the expression of a particular opinion by a person of higher status or greater seniority was confirmed by echoing statements from others who spoke later in a sequence. There is no space here for this fine-grained analysis. The data are to be made available on line, and can be subjected to further finer grained analysis. But we think the reader will benefit from an indication of some general patterns in the present paper. We have now tried to make this clearer in the paper without freighting it with more explanatory detail than is actually needed to understand the current descriptive account. |
| The quotation used at line 233-234 doesn’t seem to relate to the point being made directly above insofar as it seems to focus more on community referral than on providing care to patients inside a CCC. | CCC were more accessible and rapid sources of diagnosis. “Knowing your status” was a great concern to community members. Thus it was seen as advantageous to have a CCC as a diagnostic facility. And even if the diagnosis came back positive the first admission would have the family standing close by. We think the quotation is relevant to understanding how CCCs were viewed by communities. They were not viewed solely as care centres, but as centres of reassurance. |
| As the outbreak progressed, ETCs changed their practice and found ways to provide opportunities for interaction between patients and family members. The authors should acknowledge that at lines 236-239, and provide references. | Acknowledged and text amended |
| Line 244: What is meant by “villagers are committed to a lifetime of visits”? | To those to whom they are linked by kinship, marriage or patronage. Sentence now amended. |
| Lines 243-248 – refs needed to support descriptions of care practice in rural SL. | Reference added to Ferme 2001 |
| How do the authors interpret the fact that participants from CCC and control villages seemed to think that there was more chance of family visits at the CCC, despite the fact that control villagers would not have had experience of a CCC? | Referral [control] villages did have experience of CCC, since that was the one to which they took any patients, even though it was in a neighbouring village. |
| Lines 295-297: unclear what the significance is of explaining what was expected from FGDs unless clear data are shown to illustrate how these expectations were confounded. | This claim is made good in the following paragraphs |
| Lines 297-300: it would be interesting if the authors explored in more depth the conflict between the two types of social ordering they identify as critical in the SL Ebola period.  Were there differences, across any of the themes, depending on whether data collection took place in a control or CCC village?  If not, what do the authors posit as reasons for that?  If the overall hypothesis is that the CCC was a more acceptable intervention then you might expect to see differences in the way they were discussed | This is explored in more detail in Bulte et all ref. 12  Our finding is that differences were small.  That referral villages benefited from CCCs as much as villages where the CCC was located, except that some people in referral villages would have liked to see payroll and other tangible benefits more firmly located in their own community.  This is our conclusion, not an hypothesis. The control village was not a ‘blind control’ nor did it have an alternative Ebola facility. |
| Line 309: unclear what is meant here | Revised the sentence to clarify it. |
| Line 316-317: in what way do these words ‘hint at a stand-off’? | Because the statement rhetorically proclaims that if you let the government burial teams have the corpse the family will never see it at the funeral. |
| The authors over-rely on the distinction between hierarchical and enclave social ordering as a means of theoretically grounding their findings. How much evidence is there that rural Sierra Leonean villages are ordered as ‘enclaves’, and is this sufficient as a means to describe all the phenomena identified during this research?  Might the experience of an emergency have affected local ways of viewing and constructing ordering?  Is there evidence from other similar non-emergent settings or emergencies that the authors could discuss to explore this further? | This part of the argument was stripped from the paper for reasons of length. It is much more fully expounded both in Bulte et al. [reference 12, cited] and the first author’s forthcoming thesis.  It might, but that requires further research.  There is a large body of supporting research discussed in the two sources just mentioned and in a book on neo-Durkheimian social theory by 6 & Richards 2017. |
| Lines 327-333 is there necessarily a tension between EVD death reporting requirements and the traditional systems? What would prevent families from reporting in the traditional way while also alerting the appropriate authorities? | The point is that it is not considered proper to allow the information concerning death pass around unless it is communicated by those with the right to make such an announcement. So if the traditional rules were followed the Ebola response teams would not have been told for some days. |
| Lines 341-345 – if phones or radio announcements are ‘widely used’ in SL, why was it a surprise that these were reported as acceptable ways of receiving the news of the loss of a loved one? | In the light of the previous sentence about face-to-face reporting. In other words, tradition is more flexible than the word leads one to expect. We have rephrased to avoid any misunderstanding. |
| When reporting results that are necessarily embedded in policy or implementation changes, it would be helpful if the authors could make it clearer when events being reported are based on literature or an events timeline, or based on participant reports e.g. line 364 “some families offered land as a gesture of community solidarity” – is this based on other evidence or does it emanate from the data collected for this study? This point stands throughout much of the results section e.g. for more clarity on which evidence source supports a given conclusion. | We refer only to data offered in support of the paper, but much of it is now in the online appendix, due to considerations of length. We have tried to take care of the referee’s concerns by clarifying that our remarks are limited to data offered as part of the paper, and where needed we have clarified some statements. |
| **Discussion** |  |
| Lines 385-389: is this example from the data collected for this study or from other evidence? Not clear and needs a quote or references. | This is indeed from data and phrased summarize by the authors in a way common in discussion sections. Text amended to clarify. |
| We know that IPC practice at some CCCs was poor, and that staff there struggled with poor supplies, not receiving payroll or hazard pay, and were often dependent on handouts from the community to provide food and other basics – the authors should acknowledge this and some of the other challenges associated with the CCC approach, even if the overall conclusion is that they were a positive intervention from the community engagement perspective. | This is covered elsewhere in the text. |
| Lines 418-420: again, there were challenges with CCCs and to my knowledge there is published evidence suggesting that nosocomial transmission might have been exacerbated at these centres. The authors should make this clear and provide references. | This is acknowledged earlier in the text. |
| Lines 421 and elsewhere: the discussion of community perceptions of burial processes should acknowledge that the burials intervention changed throughout SL over the course of the outbreak and moved towards a ‘safe and dignified’ model in which families were invited to observe and attend. Presenting burials from CCCs as the only location where this approach was utilised is misleading.  Perhaps the authors could include a timeline showing policy developments for key interventions across the outbreak and explore the specific phenomena emanating from their data collection in light of the wider context, rather than in isolation? | That is not what we argue, but we will safeguard against that misreading. |
| Lines 443-445 – the evidence presented does not suggest that land acquisitions were as contentious an issue as suggested. Could the authors add some illustrative quotes and explore this further in the associated results section? | We do not agree with this reading of the evidence we present. We doubt, however, that adding one or two more illustrative quotes would resolve the issue. It requires a much more extensive treatment. The first author addresses the issue in her forthcoming thesis. |
| Lines 447-465: this is the first time knowledge brokering is mentioned. If the authors think this is a critical theme (and other published evidence does indeed suggest it might be), could they explore this further in the results section before introducing it in the discussion? Again, some references from other literature would help strengthen the argument about the importance of e.g. traditional healers as knowledge brokers in SL. | We think it requires extensive further research, but have taken the opportunity to flag the topic better in the discussion. |
| Line 468: This is highly contested and definitely needs supportive referencing. There are many factors that might have affected earlier reporting, and highlighting CCCs as a critical factor should be done with caution and evidentiary support. | We cite evidence, and have made what we say appear more cautious. We now say only that there is some evidence from one study, in addition to what people told us in fcus groups (that they were more able and more inclined to report cases quickly to CCCs). |
|  |  |

**Reviewer #3:**

| **Comments**  **Methods** | **Responses** |
| --- | --- |
| The methods seem clear however somewhat confusing. the statement that "We have chosen these four topics as they reveal differences between villagers and (inter)national health agencies most prominently" -- this is not clearly explained. | It has been more clearly explained. |
| Also unclear the time between CCC set up and data collection (feb 2015 stated, but not clear what the gap between set up was) | Further information added to section on methods. |
| explanation of CCCs remains very limited - as if there is only one type of CCC, when in fact there were multiple models (several reports in fact simply explaining the multitude of types and variations) - unclear how this was accounted for. It could at least be mentioned as something that wasnt specified or looked at (but should be acknowledged). | It is now mentioned in these terms. |
| the SDB aspect is a bit confusing - the analysis is on CCCs. the topics (four) that were the point of this paper were reportedly because they most frequently arose in discussions. However, on line 284 it states "Focus groups were asked to discuss the impact of burial regulations introduced to break Ebola infection chains" -- i'm then not clear on the link with CCCs. Unless the link was different aspects required to break transmission? (ex. SDBs, CCCs etc). I think the SDB aspect is not so clear in terms of how this comes out/ is linked to CCCs. Following this, the analysis is better explained, however I feel that this should be better explained earlier / later on (ex. 323-325 is not related to CCCs) | We do not agree. CCCs had their own SDBs or deployed one of the teams run under the authority of the DERC. Again, we wish to emphasise that our focus is a community perspective, where sickness, treatment, death and burial are inseparable, and not something divided into separate organizational compartments. Communities saw the SDBs as an integral aspect of the CCC “experience” since they commented frequently on how much better it was when the management of the CCC phoned them to explain that their patient had died and that the burial team would now be deployed, compared to the earlier sudden arrival of an unannounced burial team. We feel that our account is ethnographically correct, in that the local CCC with socially-known personnel, e.g a well-regarded CHO, served as a welcome “broker” in a distressing situation. However, line 289 is misleading and has been amended, to make it clear that FGDs discussed SDBs at their own insistence not because we asked them to. |
| As well, the paper implies that all CCCs were supporting SDBs, which I do not believe was the case | Changed to “some”. |
| **Results** |  |
| unclear about the burials in the introduction this doesn’t come up as one of the 4 themes, but appears in summary and introduction | We hope that this is now much clearer in the section on burials and reporting death. |
| **Discussion** |  |
| conclusion and discussion is the strongest component however doesnt link to the complicated methodology and coding/ analysis.  Is there a possibility to reduce the complexity of analysis? the scales seem less pertinent in the discussion/ conclusion or at least not linked. however this section is the most interesting / useful. | We depend on a willingness to look at the tables and data supplied with the paper. Not sure we can do much about this, other than via the extensive clarifications we have added to the text, including pointers to the data, taken up as supporting information. Otherwise the paper would be much longer |
| **Textual changes** |  |
| Some sentences are not clear (example "Nursing staff triaged sick persons when they reported." --> incomplete sentence) similarly "Others – due to lack of availability of ambulances – were cared for in the CCC and discharged if they survived" -- which "others" - also unclear.   line 339 - similar sentence which is unclear "CCC may not have been an agreed policy"   needs some general revision for flow  the inclusion of burials in the summary and introduction is not as clear as the rest of the results shared in intro/summary and should be better explained.    would be useful to have some recommendations for HOW these conclusions could be considered and what would be challenging/ not generally application. there is no additional discussion of considerations for culture/ leaders/ community roles in terms of recommendations/ considerations in current/ future outbreaks | Sentence is not needed and has been deleted. Others clarified.  This has been edited.  Yes, agreed, and this has been done, following suggestions from other reviewers  That section has been placed elsewhere and clarified.  We like this suggestion, but hesitate since we have already added quite considerable to the paper in taking other suggestions into account. |
